# CXCL12-induced VLA-4 activation is impaired in trisomy 12 chronic lymphocytic leukemia cells: a role for CCL21

**DOI:** 10.18632/oncotarget.3660

**Published:** 2015-03-26

**Authors:** Sylvia Ganghammer, Evelyn Hutterer, Elisabeth Hinterseer, Gabriele Brachtl, Daniela Asslaber, Peter William Krenn, Tamara Girbl, Petra Berghammer, Roland Geisberger, Alexander Egle, Antonella Zucchetto, Anna Kruschinski, Valter Gattei, Alexandre Chigaev, Richard Greil, Tanja Nicole Hartmann

**Affiliations:** ^1^ Laboratory for Immunological and Molecular Cancer Research, 3rd Medical Department with Hematology, Medical Oncology, Hemostaseology, Infectious Diseases and Rheumatology, Oncologic Center, Paracelsus Medical University Salzburg, Austria; ^2^ Salzburg Cancer Research Institute, Salzburg, Austria; ^3^ Clinical and Experimental Onco-Hematology Unit, Centro di Riferimento Oncologico, Aviano, Italy; ^4^ NOXXON Pharma AG, Berlin, Germany; ^5^ Department of Pathology and Cancer Center, University of New Mexico, Albuquerque, NM, USA

**Keywords:** trisomy 12, homing, CD49d, CXCR4, CCR7

## Abstract

Homing to distinct lymphoid organs enables chronic lymphocytic leukemia (CLL) cells to receive pro-survival and proliferative signals. Cytogenetic aberrations can significantly affect CLL cell compartmentalization. Trisomy 12 (tri12) defines a CLL subgroup with specific clinical features and increased levels of the negative prognostic marker CD49d, the α4-subunit of the integrin VLA-4, which is a key regulator of CLL cell homing to bone marrow (BM). Chemokine-induced inside-out VLA-4 activation, particularly via the CXCL12-CXCR4 axis, increases the arrest of various cell types on VCAM-1 presenting endothelium. Here, we demonstrate that high CD49d expression in tri12 CLL is accompanied by decreased CXCR4 expression. Dissecting functional consequences of these alterations, we observed that tri12 CLL cell homing to murine BM is not affected by CXCR4-CXCL12 blockage using AMD3100 or olaptesed pegol/NOX-A12. In line, CCL21-CCR7 rather than CXCL12-CXCR4 interactions triggered VLA-4-mediated arrests of tri12 CLL cells to VCAM-1 under blood flow conditions. Concordantly, in real-time kinetic analyses we found CCL21 but not CXCL12 being capable to induce inside-out VLA-4 conformational changes in this CLL subgroup. Our results provide novel insights into the peculiar clinico-biological behaviour of tri12 CLL and emphasize its specific chemokine and integrin utilization during pathophysiologically and therapeutically relevant interactions with the microenvironment.

## INTRODUCTION

The interactions between chronic lymphocytic leukemia (CLL) cells and non-malignant accessory cells within lymphoid organs are important for disease progression and relapses after therapy by providing signals for CLL cell survival and proliferation [1-3]. To receive these signals, circulating CLL cells have to extravasate into the bone marrow (BM) and secondary lymphoid tissues in a fine-tuned sequential manner. Against previously held concepts that this circulation occurs homogenously and constitutively, the homing to distinct lymphoid tissues is differentially regulated [4], and cytogenetic aberrations can have significant effects on the compartment distribution of CLL cells [5, 6]. Driving leukemic cells out of these lymphoid organs is a promising therapeutic target [7, 8]. Therefore, more detailed knowledge on the specific homing mechanisms of different CLL subgroups is required.

The cytogenetic aberration trisomy 12 (tri12) is associated with high rates of CLL cell proliferation *in vitro* and *in vivo* [9, 10], disease progression [11], lymph node (LN) involvement [6, 12] and Richter's transformation [13].

We recently demonstrated that CD49d, the alpha subunit of the VLA-4 (alpha4/beta1; CD49d/CD29) integrin, is expressed at unexpected high levels by CLL cells harboring tri12 [14]. VLA-4 is a key molecule of CLL cell homing to BM [4, 15], and CD49d has emerged as the strongest flow cytometry-based negative prognostic marker in CLL [16]. During BM homing, the heterodimer VLA-4 is thought to be activated via inside-out signaling mediated by the chemokine receptor CXCR4 and its ligand CXCL12 [17]. Mechanistically, binding of endothelially displayed CXCL12 to CXCR4 triggers several intracellular signaling events that induce a rapid conformational change of VLA-4 from a low to a high affinity state for its ligand vascular cell adhesion molecule-1 (VCAM-1), which is present on the endothelial surface [18]. These high affinity VLA-4-VCAM-1 interactions result in the immediate cell arrest of various cell types and subsequent extravasation into the tissue [17, 19]. Notably, VLA-4 is the major CD49d containing integrin in CLL, allowing CD49d measurements as a surrogate for VLA-4 [4].

Here, we investigated the interplay of CXCR4 and VLA-4 during extravasation of tri12 CLL cells compared to those not harboring this aberration (no tri12), finding the chemokine CXCL12 being unable to support BM homing and to efficiently activate VLA-4 in contrast to the successful VLA-4 activation by the LN homing chemokine CCL21.

## RESULTS

### CXCR4 expression is reduced in tri12 CLL

**Figure 1:**
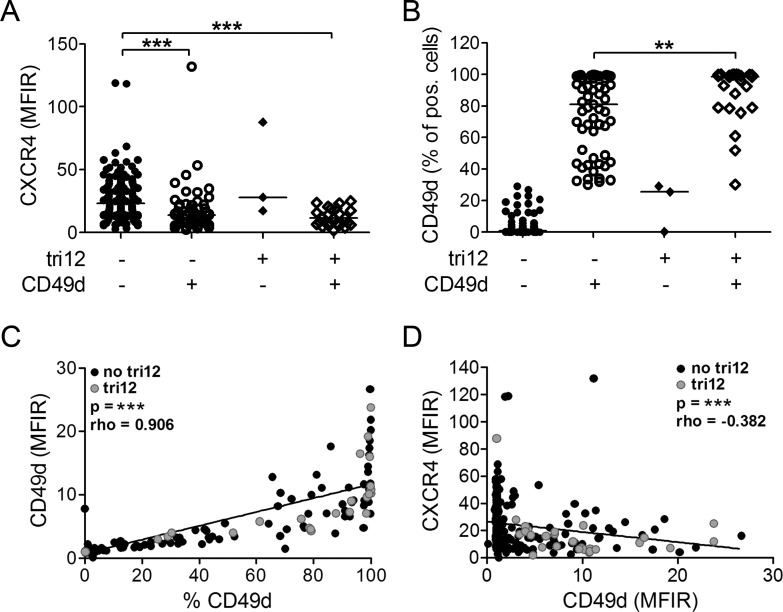
CXCR4 and CD49d expression of tri12 and no tri12 CLL cells Flow-cytometric determination of (**A**) CXCR4 MFIR values and (**B**) CD49d expression (% positive cells) in CLL cases split into four groups according to the presence of tri12 and CD49d (tri12−/CD49d−: n = 142, tri12−/CD49d+: n = 57, tri12+/CD49d−: n = 3, tri12+/CD49d+: n = 24). Scatter plots, the middle lines indicate the median. CD49d− subgroups are defined by expression on <30%, CD49d+ subgroups by expression on ≥30% of the CLL cells. (**C**) Correlation between the percentage of CD49d+ cells and the corresponding CD49d MFIR values. (**D**) Correlation (Spearman's test) between the MFIR values of CD49d and CXCR4 (n = 226).

To investigate the contribution of CXCL12-CXCR4 signals to VLA-4 dependent BM homing of tri12 versus no tri12 CLL cells, we first compared CXCR4 expression in a cohort of 226 CLL samples expressing or not expressing the VLA-4 rate limiting subunit CD49d, according to the well established prognostic cutoff of 30% positive cells [16, 22], and bearing or not bearing tri12 (Supplemental Table 1). CXCR4 expression, measured as mean fluorescence intensity ratios (MFIR), was significantly lower in CD49d+ subgroups than in CD49d− subgroups, the lowest levels being found in CD49d+ tri12 samples (Figure 1A). Notably, this reduction did not result in decreased fractions of CXCR4+ cells but was a global reduction of CXCR4 expression intensity on all CLL cells within the sample (Supplemental Figure 1). CD49d expression (% positive cells) was highest in CD49d+ tri12 samples, confirming our recent data (Figure 1B) [14]. Consistently, the percentage of CD49d+ cells directly correlated with the CD49d fluorescence intensity (Figure 1C). We also observed a moderate inverse correlation of CXCR4 and CD49d expression intensities on CLL cells (Figure 1D).

### Tri12 CLL cells home to BM independently from CXCL12-CXCR4 signals

**Figure 2:**
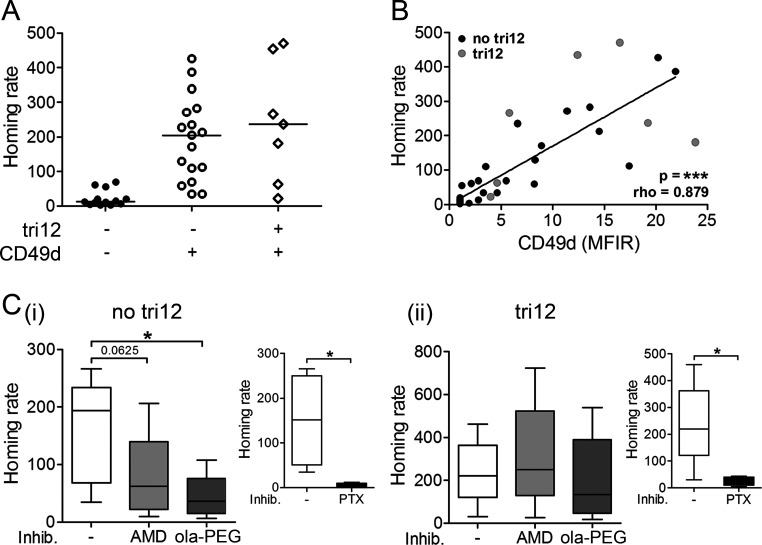
Tri12 CLL cells display CXCL12-independent BM homing (**A**) BM homing of CLL cases split into three groups according to the presence of tri12 and CD49d (tri12−/CD49d−: n = 13, tri12−/CD49d+: n = 17, tri12+/CD49d+: n = 7). Homing rates were normalized as described [4, 15]: number of human cells analyzed per 10^6^ mouse cells (total cells) per 10^6^ injected viable human target cells. Scatter plots, the middle lines mark the median. (**B**) Correlation (Spearman's test) between the homing rate and the corresponding CD49d expression (MFIR) of no tri12 (n = 27) and tri12 (n = 7) CLL cells. (**C**) BM homing rates of (i) no tri12 and (ii) tri12 CLL cells upon treatment with AMD3100, olaptesed pegol/NOX-A12 and PTX (small plots, positive control). The data represent the results of 5 different samples tested in each subgroup (PTX control no tri12: n = 4).

To study the functional consequences of these inverse CD49d and CXCR4 expression levels in tri12 CLL samples, we performed short term *in vivo* homing assays. For this purpose, we adoptively transferred human leukemic cells into NOD/SCID mice and evaluated the homing capacity of the CLL cells to BM or spleen as previously described [15]. We found slightly increased BM homing rates of tri12 compared to no tri12 cases, presumably due to their generally higher CD49d expression. However, comparing CD49d+ no tri12 and CD49d+ tri12 CLL, we detected no difference in the relative homing capacity (Figure 2A). In line, the BM homing rate of CLL cells most strongly correlated with their CD49d expression intensity (Figure 2B), suggesting CD49d the only decisive factor. All BM homing, irrespective of the presence of tri12, was dependent on functional CD49d, as demonstrated by the use of blocking anti-CD49d antibodies, confirming our previous observations (Supplemental Figure 2A) [4, 15]. Next, we analyzed the contribution of CXCL12-CXCR4 signals to CLL homing by pretreating the mice with the CXCR4 antagonist AMD3100, which competes with CXCL12 for receptor binding [23], or the anti-CXCL12 Spiegelmer® ola-PEG (also known as NOX-A12) [24], before injecting human CLL PBMCs intravenously. Both treatments resulted in a reduced homing capacity of CLL cells with a karyotype other than tri12, with a stronger and more consistent antagonistic potential of ola-PEG than AMD3100 (Figure 2C(i)). In contrast, BM homing of tri12 CLL cells was not antagonized by AMD3100 or ola-PEG, suggesting that homing of these cells occured through a mechanism independent from CXCL12-CXCR4 signaling (Figure 2C(ii)). In line, the few residual CLL cells in no tri12 samples that were able to home to BM under CXCL12-CXCR4 blockage displayed elevated CXCR4 levels, suggesting a selection or rescue mechanism occurring in this CXCR4 high subpopulation (Supplemental Figure 2B). This phenomenon was not observed in tri12 samples (Supplemental Figure 2B). However, BM homing in both no tri12 and tri12 CLL samples was abrogated by pertussis toxin (PTX), an inhibitor of Gαi proteins that are involved in the integrin inside-out activation cascade triggered by chemokines (Figure 2C, insets). Spleen homing was not reduced by CXCL12-CXCR4, CD49d or Gαi blockage (data not shown), neither in no tri12 nor in tri12 harbouring CLL cells, in line with previous data [4, 15].

In summary, we observe a crucial role of Gαi-coupled proteins but not CXCL12-CXCR4 signals in VLA-4 mediated homing of tri12 CLL cells to BM, suggesting VLA-4 activation by a Gαi-coupled mechanism independent from CXCL12.

### CXCL12 does not increase arrests of tri12 CLL cells on VCAM-1 under shear flow

**Figure 3:**
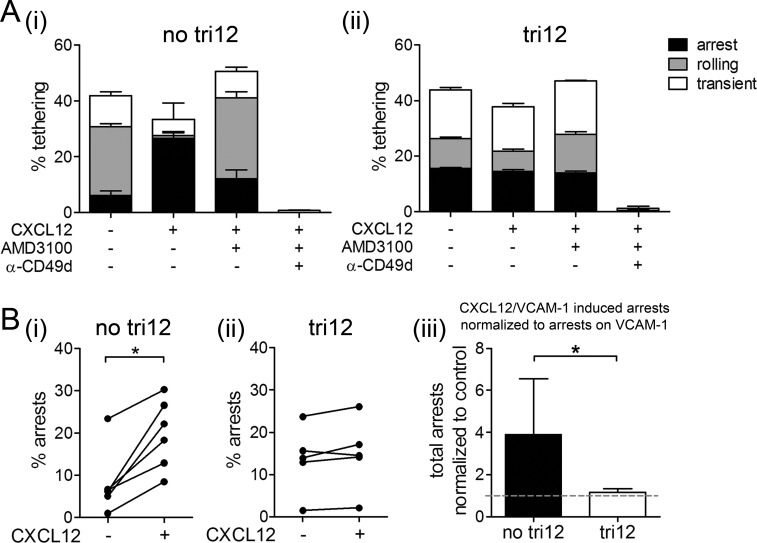
CXCL12 induces arrests of no tri12 but not of tri12 CLL cells on VCAM-1 under shear stress (**A**(i)) No tri12 and (ii) tri12 CLL cells were treated with/without AMD3100 or blocking anti-CD49d mAb and perfused for 1 min at 0.5 dyn/cm^2^ over VCAM-1 co-immobilized with CXCL12. Categories of interactions (tethers) are expressed as frequencies of cells in direct contact with the substrate. The data show one individual representative out of 5-6 independent experiments each; the columns show the mean ± SD of triplicates. (**B**) Summary and statistics of all samples: Total arrests induced by VCAM-1 and VCAM-1/CXCL12, respectively, of (i) no tri12 (n = 6) and (ii) tri12 (n = 5) CLL cells under shear stress. (iii) Total arrests to CXCL12 of no tri12 (n = 6) and tri12 (n = 5) CLL cells normalized to VCAM-1 controls; columns represent the mean ± SD.

We next used a controlled *in vitro* approach mimicking the physiological blood flow to address why tri12 CLL cells home independent from CXCL12-CXCR4 signals to BM. Under normal conditions, endothelial CXCL12 binds to CXCR4 on lymphocytes, thereby inside-out activating VLA-4, converting a low affinity binding phenotype (“rolling”) of the cells to a stable shear-resisting adhesion (“arrest”) to the blood vessels [19, 20]. Using parallel flow chambers, we perfused CD49d+ no tri12 and tri12 CLL cells over VCAM-1 or VCAM-1/CXCL12 substrates and analyzed the rates and categories of cell tethering at a single cell level by videomicroscopy. We found that VLA-4 expressed on no tri12 CLL cells could undergo CXCL12-mediated activation, inducing the arrest of CLL cells on VCAM-1 under shear stress. These chemokine-induced cell arrests on VCAM-1 were highly susceptible to CXCR4 blocking with AMD3100 (Figure 3A(i)). In contrast, tri12 CLL cases demonstrated a high ability to arrest on VCAM-1 under shear flow even in absence of the chemokine. These arrests could neither be augmented by CXCL12 nor could they be inhibited by AMD3100 (Figure 3A(ii)). As expected, all interactions were abrogated by anti-CD49d antibodies in both, no tri12 and tri12 CLL cells (Figure 3A). A significant CXCL12-induced increase in tethering was observed in all no tri12 CLL samples tested (Figure 3B(i)), whereas tri12 CLL cell tethering was not influenced by this chemokine (Figure 3B(ii)). In average, an approximately 4fold increase in arrests could be attributed to CXCL12 in no tri12 cases as compared to tri12 cases (Figure 3B(iii)).

### CXCL12 masters migration of no tri12 and tri12 CLL cells

**Figure 4:**
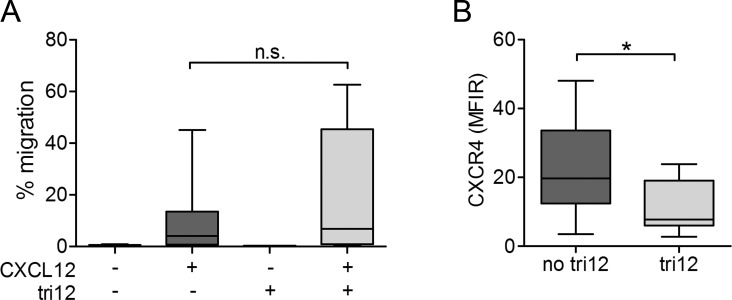
Chemotaxis of tri12 CLL towards CXCL12 is intact despite lower CXCR4 expression (**A**) Boyden chamber migration assays of no tri12 (n = 17) and tri12 (n = 7) CLL cells towards CXCL12. Each experiment has been performed in duplicates; columns show the mean ± SD of all experiments performed. (**B**) Flow-cytometric comparison of CXCR4 surface expression of no tri12 and tri12 CLL samples used in (A).

Collectively, our observations could either point to a general CXCR4 dysfunction or to a specific alteration in the CXCL12-induced VLA-4 activation cascade in tri12 CLL cells. We therefore tested the ability of no tri12 versus tri12 CLL cells to migrate towards CXCL12 in chemotaxis assays. Notably, under these shear free conditions, tri12 CLL cells tended to migrate towards CXCL12 even at higher rates than no tri12 CLL cells (Figure 4A) despite expressing lower CXCR4 surface levels (Figure 4B). Chemotaxis was markedly reduced by AMD3100, ola-PEG and PTX in both subgroups to a similar extent (Supplemental Figure 3). These findings suggest a fully functional CXCR4 receptor and an integrin-independent random motility that is not compromised in tri12 CLL cells.

### VLA-4 expressed on tri12 CLL cells is not pre-activated

VLA-4 can be present in multiple conformations with different affinity for its ligand VCAM-1. The low affinity bent state of the integrin translates into a non-adhesive resting cell, the low affinity unbent or extended state results in cell rolling, and the high affinity extended state promotes cell arrest [18]. In CLL, little is known about the characteristics of VLA-4 affinity regulation under physiological conditions.

**Figure 5:**
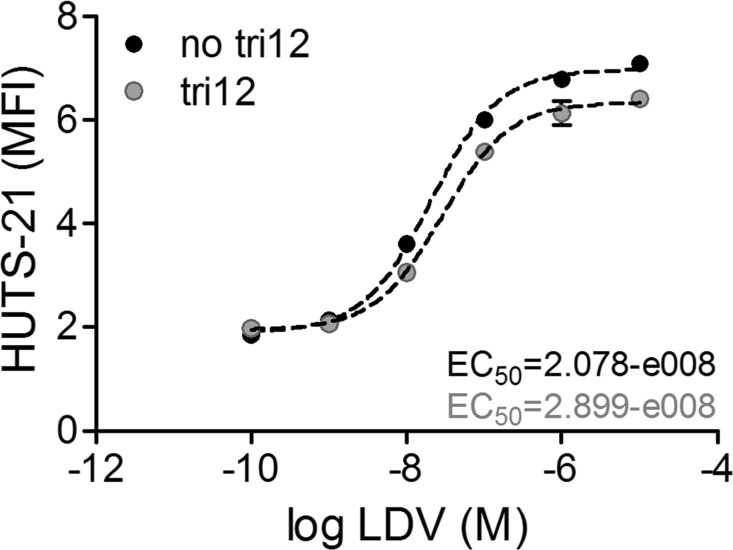
VLA-4 expressed on tri12 CLL cells is not in a pre-activated conformation Binding of HUTS-21 mAb plotted versus LDV concentration. No tri12 and tri12 CLL cells were incubated with the indicated LDV concentrations in presence of an excess of HUTS-21 mAb. The fit to the data was done using the sigmoidal dose-response equation with variable slope using GraphPad Prism software. The EC_50_ values calculated for both subgroups indicate a resting state of the VLA-4 integrin [25]. A representative experiment of 2-3 independent experiments in the tri12 (n = 3) and no tri12 (n = 2) cohort is shown. Each point represents the mean ± SD of triplicates.

Based on our observation of the strong basal interactions of unstimulated tri12 CLL cells with the VCAM-1 substrate under flow, we hypothesized a pre-activated high affinity VLA-4 conformation on these cells. Therefore, we used the conformationally sensitive anti-beta1 (CD29) antibody HUTS-21 that detects the exposed hybrid domain of the beta1 subunit. In the presence of VLA-4 ligand occupancy, absolute rates of HUTS-21 binding to activated cells are faster than the binding rates to resting cells, thus reporting the high affinity state of VLA-4 [25]. For VLA-4 ligation, we used the small molecule LDV, a peptide derived from the conserved high affinity LDV sequence of the VLA-4 binding site [21]. However, the binding kinetics of no tri12 and tri12 cells to the HUTS-21 antibody were comparable, indicating that tri12 CLL do not express pre-activated VLA-4 (Figure 5).

### The CCR7 ligand CCL21 increases arrests of tri12 CLL cells on VCAM-1

**Figure 6:**
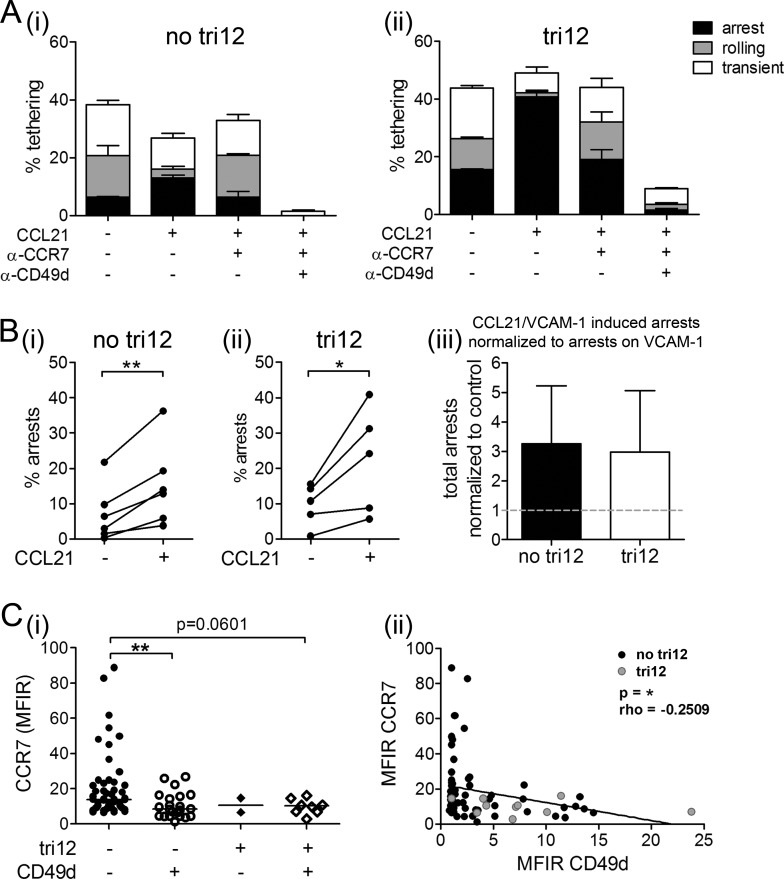
CCL21 is capable to induce arrests of tri12 CLL cells on VCAM-1 under shear stress (**A**(i)) no tri12 and (ii) tri12 CLL cells were treated with/without blocking anti-CCR7 mAb or anti-CD49d mAb and perfused for 1 min at 0.5 dyn/cm^2^ over VCAM-1 co-immobilized with CCL21. Categories of interactions (tethers) are expressed as frequencies of cells in direct contact with the substrate. The data show one individual representative out of 5-6 experiments each; the columns show the mean ± SD of triplicates. (**B**) Summary and statistics: Total arrests induced by VCAM-1 and VCAM-1/CCL21, respectively, of (i) no tri12 (n = 6) and (ii) tri12 (n = 5) CLL cells under shear stress. (iii) Summarized relative total arrests to CCL21 under shear stress of no tri12 (n = 6) and tri12 (n = 5) CLL cells normalized to VCAM-1 controls; columns indicate the mean ± SD. (**C**) Flow-cytometric determination of the (i) CCR7 MFIR values in CLL cases split into four groups according to the presence of tri12 and CD49d (tri12−/CD49d−: n = 53, tri12−/CD49d+: n = 22, tri12+/CD49d−: n = 2, tri12+/CD49d+: n = 8); Scatter plots, the middle lines indicate the median. (ii) Correlation (Spearman's test) between the MFIR values of CD49d and CCR7 (n = 85).

Next, we aimed to elucidate whether the observed VLA-4 unresponsiveness was restricted to the CXCR4-VLA-4 signaling pathway only or relevant for chemokine-induced VLA-4 activation in general. Therefore, instead of using CXCL12, we coated flow chamber slides with VCAM-1 and CCL21, a ligand to the lymph node homing receptor CCR7. In presence of CCL21, we observed increased arrests on VCAM-1 in no tri12 and tri12 CLL cells, suggesting similar VLA-4 activation upon CCR7 engagement by CCL21 in both subgroups. These CCL21-induced arrests were strongly reduced by blocking CCR7 and nearly abolished by anti-CD49d antibodies (Figure 6A). Quantification of the CCL21-induced arrests in all analyzed samples indicated comparable significant activation of VLA-4 in no tri12 and tri12 CLL samples (Figure 6B). Similar to the expression pattern of CXCR4 in the different subgroups (compare to Figure 1A), we found that CD49d+ CLL cells expressed somewhat lower CCR7 levels than CD49d− CLL cells, with a tendency to an inverse correlation of CCR7 and CD49d (Figure 6C).

### CCL21, but not CXCL12, induces an activated VLA-4 conformation on tri12 CLL cells

VLA-4-dependent adhesion is controlled by several conformational states of the integrin with reversible changes in affinity occurring within subseconds [18]. Because of this rapid kinetics, we decided for real-time assays to investigate the alterations in VLA-4 conformational states over time in tri12 versus no tri12 cases. We analyzed the kinetics of complex formation and dissociation of VLA-4 with its ligand upon stimulation with CXCL12 and CCL21, using LDV-FITC, a fluorochrome-conjugated peptide ligand to VLA-4 [21]. The binding of fluorescent LDV to VLA-4 expressed on CLL cells was cytometrically detected. Unlabeled LDV, added in a 500-fold excess, competed with LDV-FITC for VLA-4 binding and allowed the calculation of the dissociation rate constant (K_off_) as a measurement for the affinity of the receptor for its ligand. Manganese, a stimulus inducing the maximal extent of VLA-4 activation, which is not achieved under physiological conditions, was used as a technical positive control in these assays, as described [21].

In absence of any stimulation, both, no tri12 and tri12 CLL cells bound the ligand with comparable affinity and K_off_ values between 0.06-0.08 s^−1^. These values are common for unactivated VLA-4 and comparable between various cell types [21]. Likewise, upon manganese stimulation, affinities and K_off_ values were similar in both CLL subgroups (0.010-0.012 s^−1^). However, the extent of bound LDV-FITC to VLA-4 was higher in tri12 CLL upon positive control stimulation due to higher VLA-4 expression in this subgroup.

**Figure 7:**
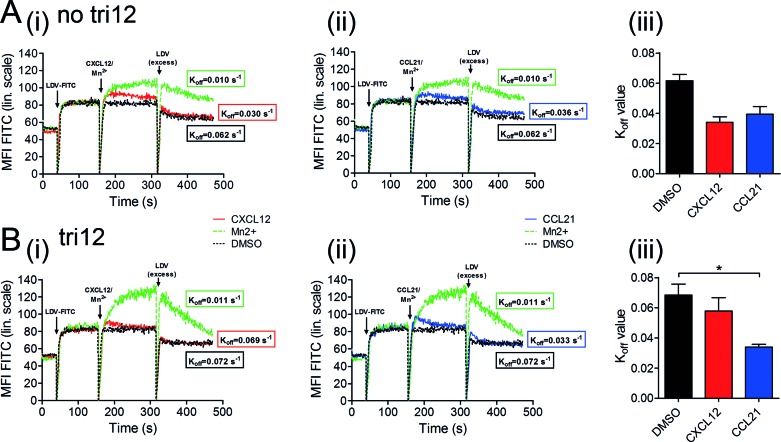
CCL21, but not CXCL12, induces an activated conformation on tri12 CLL cells In flow-cytometric real-time kinetic analysis, (**A**) no tri12 and (**B**) tri12 CLL cells were incubated with LDV-FITC and stimulated with (i) CXCL12 or (ii) CCL2, as well as Mn^2+^ or DMSO, resulting in a different equilibrium binding of LDV-FITC to VLA-4, depending on the respective integrin affinity state. Dissociation kinetics of the fluorescent LDV peptide from the cells is induced by 500-fold excess of unlabeled peptide. To obtain the dissociation rate constants (K_off_), the data were fitted to a one-phase exponential decay equation. The calculated K_off_ values indicate the VLA-4 affinity state after stimulating the cells with the three different substances; K_off_ < 0.02 s^−1^ high affinity, K_off_ > 0.06 s^−1^ low affinity. The data represent one out of 2-3 independent experiments with no tri12 (n = 2) and tri12 (n = 3) CLL cells; each experiment has been performed in duplicates. (A/B(iii)) Summary of the K_off_ values after VLA-4 activation by DMSO, CXCL12 or CCL21 in (A(iii)) no tri12 and (B(iii)) tri12 CLL cells. The data represent the mean ± SD of 2-3 experiments.

Upon stimulation with CXCL12 or CCL21, a rapidly enhanced binding of no tri12 CLL cells to the ligand was observed (Figure 7A(i, ii)). The K_off_ rates for the activation with CXCL12 and CCL21 were 0.03-0.04 s^−1^ and 0.035-0.045 s^−1^, respectively (Figure 7A(iii)), and thus comparable to that of inside-out VLA-4 activation of other cell types [21]. However, in contrast to no tri12 CLL cells, tri12 CLL cells largely failed to undergo the rapid affinity up-regulation triggered by CXCL12 stimulation, in keeping with tethering experiments (Figure 7B(i)). Dissociation rate constants were at about 0.06-0.075 s^−1^ and thus comparable to solvent controls (Figure 7B(iii)). On the other hand, CCL21 did not fail to induce VLA-4 activation in tri12 CLL cells (Figure 7B(ii)), yielding similar K_off_ values as in the no tri12 CLL subgroup (Figure 7A(ii)).

## DISCUSSION

Tri12 CLL, which is characterized by high proliferation rates [9, 10], an increased prevalence of NOTCH-1 mutations [26] and Richter's transformation [13], is the third most frequent chromosomal aberration in CLL. We recently described overexpression of the VLA-4 subunit CD49d in tri12 CLL, caused by a demethylation-dependent regulatory mechanism [14]. Having also established VLA-4 as the chief orchestrator of BM homing of CLL cells [4, 15], we here dissected its interplay with the CXCL12-CXCR4 axis, an important component of the BM homing machinery of lymphocytes and progenitor cells [27] in tri12 CLL.

We found that BM homing of tri12 CLL cells is directly dependent on their high CD49d expression but barely reliant on CXCL12-CXCR4 signals, despite a fully functional CXCR4 receptor in chemotaxis assays. CXCL12-independent BM homing has been reported by several groups. Bioactive lipids such as sphingosine-1-phosphate (S1P) [28, 29] and ceramide-1-phosphate (C1P) [30, 31], as well as several extracellular nucleotides [32, 33] can guide VLA-4 mediated extravasation of hematopoeitic stem/progenitor cells (HSPCs) into BM. However, we could not identify any of these candidates effective to activate VLA-4 in tri12 CLL (data not shown) and therefore attribute their PTX sensitive BM homing to another Gαi-coupled receptor.

Under flow, we observed strongly increased tethering of tri12 CLL cells to VCAM-1 in presence of CCL21, a ligand to the LN chemokine receptor CCR7, while CXCL12 did not affect cell arrests. In line, in real-time cytometrical analyses, we detected successful inside-out VLA-4 activation induced by CCL21 but not CXCL12. Moreover, even in absence of any stimulation, tri12 CLL cells strongly interacted with VCAM-1 under shear. Since VLA-4 expressed on tri12 CLL cells did not exhibit a pre-activated conformation, it is likely that increased VLA-4 avidity based on elevated CD49d expression contributes to these enhanced adhesive properties. Furthermore, overexpression of CalDAG-GEF1, Rap1B and RAPL in tri12 CLL [34], intracellular molecules playing a crucial role in the VLA-4 inside-out signaling cascade, may lower the threshold for VLA-4 activation by signals other than CXCL12.

An important finding is that in tri12, but not in no tri12 CLL cells, the VLA-4 inside-out activation is functionally uncoupled from the general response to CXCL12, i.e. the integrin shows no or only a weak response to the stimulus while the chemotactic response is retained. This decreased sensitivity of VLA-4, which can be bypassed by incubation with Mn^2+^, cannot be related to CD49d overexpression and reduced CXCR4 receptor density only (Supplemental Figure 4). Although we cannot formally exclude that during *in vivo* homing high CD49d levels can partially compensate for decreased CXCR4 receptor densities in tri12 CLL, *in vitro* assays argue that these expressional CXCR4 differences are not decisive for the extent of CXCL12-induced arrests under shear flow (Supplemental Figure 4). Moreover, chemotaxis of tri12 CLL towards CXCL12 is intact despite their lower CXCR4 expression (Figure 4). We therefore propose a restricted alteration in the CXCR4-VLA-4 signaling cascade in tri12 CLL cells rather than a CXCR4 expression-based phenomenon.

Notably, disproportionate CXCL12 responsiveness and CXCR4 expression in B cells is long known [35], and upon BCR stimulation, increased chemotaxis of CLL cells towards CXCL12 despite decreased CXCR4 expression has been observed [36, 37]. Low CXCR4 expression is commonly observed in activated B cells and is part of the centrocyte phenotype in germinal centers [38, 39]. In CLL, low CXCR4 indicates an enrichment of recently divided tumor cells that emigrated from lymphoid tissues [40]. Consequently, we assume that the low CXCR4 expression in tri12 CLL is a manifestation of an activation phenotype or BCR stimulation, linked to the lymphoid microenvironment rather than driven by the tri12 subclone itself, which is also consistent to our recent observation of high active NF-kB in these cells [9].

Riches and colleagues recently postulated that tri12 CLL display enhanced CXCL12-induced polarization and motility on VCAM-1 under shear free conditions [34]. It is important to note that these observations are not controversial to our data as the assays used in their study mimic VLA-4 independent interstitial motility rather than VLA-4 dependent BM homing and can be compared to our chemotaxis assays. In T cells, motility triggered by the chemokines CCL21 and CXCL12 does not require functional integrins, even in presence of integrin ligands [41, 42]. Thus, from the data presented in the recent study [34], the specific contribution of VLA-4 to the observed CXCL12-induced polarization and motility of tri12 CLL cells remains elusive.

Riches and colleagues also suggested that β2-integrin (CD18) expression in tri12 CLL is modulated by the NOTCH1 mutation status [34], implying the possibility of a similar crosstalk with VLA-4. An association of NOTCH1 mutations with the tri12 karyotype in CLL is well established [26, 43-46]. Consistent to the previous studies [14, 34], we observed the expected augmented rate of the most common NOTCH1 mutation in the tri12 cohort (Supplemental Table 2) but could not identify a direct link between mutated NOTCH1 and altered CD49d expression or VLA-4 function. In a previous report, NOTCH1 mutations have been identified as the most common lesions occurring in Richter syndrome (RS) [47], thus closing the loop to the frequent presence of tri12 in this rare transformation from CLL into an aggressive lymphoma. Since the proportion of tri12 CLL cells in LNs is higher than in BM or peripheral blood [6] and tri12 is frequent in small lymphocytic lymphoma [48] and RS [13] with high lymphoid predisposition, we hypothesize that these cells have an enhanced capability to home to LNs. The preference of CCR7 over CXCR4 signals during VLA-4 mediated extravasation, which we observed, may offer a biological explanation for these characteristics. Notably, an abbreviated redistribution lymphocytosis during Ibrutinib treatment has been recently observed in tri12 CLL patients [49]. Thus, the observation that tri12 CLL use a differential mechanistic repertoire to localize in lymphoid organs is likely not only mechanistically but also therapeutically meaningful.

In conclusion, we demonstrated that tri12 CLL cells home independently from the CXCL12-CXCR4 axis but dependent on VLA-4 to BM. We observed that VLA-4 expressed on these cells is predominantly sensitive to CCR7 instead of CXCR4 signals, which implies pathophysiological consequences due to altered and presumably pronounced extravasation into LN microenvironments. In consequence, our data offer a mechanistical explanation for the peculiar clinical and biological characteristics of tri12 CLL on basis of their specific migratory properties.

## MATERIALS AND METHODS

### Ethics

Blood samples of CLL patients were taken upon written informed consent (Ethics committee approval Salzburg: 415-E/1287/4–2011, 415-E/1287/8–2011).

All experiments involving mice were performed under approval of the Austrian Animal Ethics Committee (BMWF-66.012/0011-II/10b2009, BMWF-66.012/0004-II/3b/2014).

### Patient samples and cell preparation

Blood samples were obtained from CLL patients who were chemonaive or had not received chemotherapy during the last six months at the 3rd Medical Department Salzburg. Peripheral blood mononuclear cells (PBMCs) were isolated by density gradient centrifugation and freshly used or viably frozen in FCS plus 10% DMSO for storage in liquid nitrogen. Freshly isolated or thawed cells were cultured in RPMI-1640 supplemented with 10% FCS and antibiotics. For functional assays, CLL cells were enriched untouched to ≥ 98% purity (EasySep, Stem Cell Technologies).

### Flow cytometry

CD49d, CXCR4 and CCR7 expression was routinely measured in whole blood samples from CLL patients and prior to each functional experiment in PBMCs. CLL cells were defined as CD5/CD19 double positive lymphocytes. Viable cells were identified as Annexin-V/7-aminoactinomycin (7-AAD) negative cells.

### Antibodies and reagents

Fluorochrome-labeled monoclonal antibodies (mAb) were purchased from BD, eBioscience and Beckman Coulter. Chemokines and integrin ligands were supplied by R&D Systems. For blocking experiments, AMD3100 (Sigma-Aldrich), olaptesed pegol (ola-PEG)/NOX-A12 (NOXXON Pharma), anti-CCR7 mAb clone 150503 (R&D) and anti-CD49d mAb clone HP2.1 (Abcam) were used. All other reagents and chemicals were purchased from Sigma-Aldrich. The VLA-4 specific ligand 4-((*N'*-2-methylphenyl)ureido)-phenylacetyl-L-leucyl-L-aspartyl-L-va-lyl-L-prolyl-L-alanyl-L-alanyl-L-lysine (LDV) and its FITC-conjugated analog (LDV-FITC) were synthesized at Commonwealth Biotechnologies.

### *In vivo* short term homing

CLL PBMCs were thawed and incubated overnight with/without pertussis toxin (PTX, 100 ng/ml). 5-15 × 10^6^ PBMCs were injected into NOD/SCID mice (Charles River Laboratories) that were either untreated or pretreated with AMD3100 (5 mg/kg, subcutaneous) or ola-PEG (20 mg/kg, intravenous) for 1 hour prior to injection. After 3 hours, mice were sacrificed, human CLL cells present in the murine BM, spleen and peripheral blood were cytometrically detected using specific anti-human antibodies, and the homing rate was calculated as previously described [4, 15]. Viability, CD49d and CXCR4 expression on CLL cells was cytometrically determined directly prior to injection as well as after BM homing.

### Shear flow assay

Shear flow assays were performed as previously described [4, 20]. 6-channel μ-slides (Ibidi) were coated with protein A and VCAM-1/Fc and co-immobilized with/without CXCL12 at 4°C overnight, washed, and blocked with 2% human serum albumin. Isolated CD49d+ CLL cells were incubated with/without AMD3100 (5 μM) or anti-CD49d mAb (1 μg/ml) for 10 min. The cells were perfused in the flow chamber, allowed to accumulate at sub-physiological shear stress (0.5 dyn/cm^2^) to the coated substrates, and then subjected to physiological shear stress (2 dyn/cm^2^). The entire perfusion period was recorded and digitalized. Analysis of the video-recorded segments was done using a custom designed image analysis software (Wimasis). Frequencies of adhesive categories were determined as percentages of cells flowing directly over the substrates. All experiments were performed at 37°C.

### Chemotaxis

Chemotaxis assays were performed using transwell plates with 5 μm pores (Corning Costar). CLL PBMCs were preincubated for 1 hour at 37°C with/without AMD3100 (5 μM) or PTX (100 ng/ml). CXCL12 (100 ng/ml) with/without ola-PEG (100 nM) was added to the lower chamber. 5 × 10^5^ PBMCs were placed in the upper wells. After 2 hours, migrated CLL cells were cytometrically counted using Flow Count Fluorospheres (Beckman Coulter) and anti-CD5/CD19 antibodies.

### Determination of VLA-4 conformation

Isolated CD49d+ CLL cells (1 × 10^6^ cells/ml) were solved in RPMI-1640 containing 25 nM HEPES, pH 7.4. Purity, viability and CXCR4, CCR7, CD49d, and CD29 expression were cytometrically determined prior to each experiment. Cells were incubated with the indicated concentrations of LDV in the presence of an excess of anti-CD29 mAb (clone HUTS-21) for 30 min at 37°C. Subsequently, the mean fluorescence intensity (MFI) of labeled HUTS-21 mAb was cytometrically determined.

### Real-time kinetic analysis of VLA-4 affinity after activation

Kinetic analysis of binding and dissociation of the LDV-FITC probe to VLA-4 was performed as previously described [21]. VLA-4 affinity on isolated CD49d+ CLL cells was cytometrically analyzed for up to 800 s at 37°C. Upon establishing an autofluorescence baseline for 30-60 s, 4 nM LDV-FITC was added and data were acquired for further 100 s before chemokine (100 ng/ml), Mn^2+^ (1 mM) or DMSO was added. Upon further 150 s, samples were treated with an excess (1 μM) of unlabeled LDV probe, and the dissociation of LDV-FITC was followed for further 150 s. The resulting data were converted to MFI versus time using the FCSQuery software (Dr. B. Edwards, University of New Mexico, Albuquerque, NM, USA).

### Statistical analysis

Statistical analysis was performed using GraphPad Prism 5.02. All data were tested for normal distribution. Normally distributed data were compared using t-tests (paired or unpaired), nonparametric data using Mann Whitney test (unpaired) or Wilcoxon high rank test (paired analysis). Results were considered statistically significant when p < 0.05. Significances are marked with asterisks (p < 0.05 = *, p < 0.01 = **, p < 0.001 = ***).

## SUPPLEMENTARY MATERIAL FIGURES AND TABLES







## References

[1] Caligaris-Cappio F, Bertilaccio MT, Scielzo C (2014). How the microenvironment wires the natural history of chronic lymphocytic leukemia. Semin Cancer Biol.

[2] Deaglio S, Malavasi F (2009). Chronic lymphocytic leukemia microenvironment: shifting the balance from apoptosis to proliferation. Haematologica.

[3] Caligaris-Cappio F, Ghia P (2008). Novel insights in chronic lymphocytic leukemia: are we getting closer to understanding the pathogenesis of the disease?. J Clin Oncol.

[4] Hartmann TN, Grabovsky V, Wang W, Desch P, Rubenzer G, Wollner S, Binsky I, Vallon-Eberhard A, Sapoznikov A, Burger M, Shachar I, Haran M, Honczarenko M, Greil R, Alon R (2009). Circulating B-cell chronic lymphocytic leukemia cells display impaired migration to lymph nodes and bone marrow. Cancer Res.

[5] Dohner H, Stilgenbauer S, James MR, Benner A, Weilguni T, Bentz M, Fischer K, Hunstein W, Lichter P (1997). 11q deletions identify a new subset of B-cell chronic lymphocytic leukemia characterized by extensive nodal involvement and inferior prognosis. Blood.

[6] Liso V, Capalbo S, Lapietra A, Pavone V, Guarini A, Specchia G (1999). Evaluation of trisomy 12 by fluorescence in situ hybridization in peripheral blood, bone marrow and lymph nodes of patients with B-cell chronic lymphocytic leukemia. Haematologica.

[7] Byrd JC, Furman RR, Coutre SE, Flinn IW, Burger JA, Blum KA, Grant B, Sharman JP, Coleman M, Wierda WG, Jones JA, Zhao W, Heerema NA, Johnson AJ, Sukbuntherng J, Chang BY (2013). Targeting BTK with ibrutinib in relapsed chronic lymphocytic leukemia. The New England journal of medicine.

[8] Furman RR, Sharman JP, Coutre SE, Cheson BD, Pagel JM, Hillmen P, Barrientos JC, Zelenetz AD, Kipps TJ, Flinn I, Ghia P, Eradat H, Ervin T, Lamanna N, Coiffier B, Pettitt AR (2014). Idelalisib and rituximab in relapsed chronic lymphocytic leukemia. The New England journal of medicine.

[9] Hutterer E, Asslaber D, Caldana C, Krenn PW, Zucchetto A, Gattei V, Greil R, Hartmann TN (2014). CD18 (ITGB2) expression in chronic lymphocytic leukaemia is regulated by DNA methylation-dependent and -independent mechanisms. British journal of haematology.

[10] Ciccone M, Agostinelli C, Rigolin GM, Piccaluga PP, Cavazzini F, Righi S, Sista MT, Sofritti O, Rizzotto L, Sabattini E, Fioritoni G, Falorio S, Stelitano C, Olivieri A, Attolico I, Brugiatelli M (2012). Proliferation centers in chronic lymphocytic leukemia: correlation with cytogenetic and clinicobiological features in consecutive patients analyzed on tissue microarrays. Leukemia.

[11] Rossi D, Spina V, Bomben R, Rasi S, Dal-Bo M, Bruscaggin A, Rossi FM, Monti S, Degan M, Ciardullo C, Serra R, Zucchetto A, Nomdedeu J, Bulian P, Grossi A, Zaja F (2013). Association between molecular lesions and specific B-cell receptor subsets in chronic lymphocytic leukemia. Blood.

[12] Tsimberidou AM, Keating MJ (2005). Richter syndrome: biology, incidence, and therapeutic strategies. Cancer.

[13] Chigrinova E, Rinaldi A, Kwee I, Rossi D, Rancoita PM, Strefford JC, Oscier D, Stamatopoulos K, Papadaki T, Berger F, Young KH, Murray F, Rosenquist R, Greiner TC, Chan WC, Orlandi EM (2013). Two main genetic pathways lead to the transformation of chronic lymphocytic leukemia to Richter syndrome. Blood.

[14] Zucchetto A, Caldana C, Benedetti D, Tissino E, Rossi FM, Hutterer E, Pozzo F, Bomben R, Dal Bo M, D'Arena G, Zaja F, Pozzato G, Di Raimondo F, Hartmann TN, Rossi D, Gaidano G (2013). CD49d is overexpressed by trisomy 12 chronic lymphocytic leukemia cells: evidence for a methylation-dependent regulation mechanism. Blood.

[15] Brachtl G, Sahakyan K, Denk U, Girbl T, Alinger B, Hofbauer SW, Neureiter D, Hofbauer JP, Egle A, Greil R, Hartmann TN (2011). Differential bone marrow homing capacity of VLA-4 and CD38 high expressing chronic lymphocytic leukemia cells. PLoS One.

[16] Bulian P, Shanafelt TD, Fegan C, Zucchetto A, Cro L, Nuckel H, Baldini L, Kurtova AV, Ferrajoli A, Burger JA, Gaidano G, Del Poeta G, Pepper C, Rossi D, Gattei V (2014). CD49d is the strongest flow cytometry-based predictor of overall survival in chronic lymphocytic leukemia. J Clin Oncol.

[17] Peled A, Kollet O, Ponomaryov T, Petit I, Franitza S, Grabovsky V, Slav MM, Nagler A, Lider O, Alon R, Zipori D, Lapidot T (2000). The chemokine SDF-1 activates the integrins LFA-1, VLA-4, and VLA-5 on immature human CD34(+) cells: role in transendothelial/stromal migration and engraftment of NOD/SCID mice. Blood.

[18] Chigaev A, Sklar LA (2012). Aspects of VLA-4 and LFA-1 regulation that may contribute to rolling and firm adhesion. Front Immunol.

[19] Grabovsky V, Feigelson S, Chen C, Bleijs DA, Peled A, Cinamon G, Baleux F, Arenzana-Seisdedos F, Lapidot T, van Kooyk Y, Lobb RR, Alon R (2000). Subsecond induction of alpha4 integrin clustering by immobilized chemokines stimulates leukocyte tethering and rolling on endothelial vascular cell adhesion molecule 1 under flow conditions. J Exp Med.

[20] Hartmann TN, Grabovsky V, Pasvolsky R, Shulman Z, Buss EC, Spiegel A, Nagler A, Lapidot T, Thelen M, Alon R (2008). A crosstalk between intracellular CXCR7 and CXCR4 involved in rapid CXCL12-triggered integrin activation but not in chemokine-triggered motility of human T lymphocytes and CD34+ cells. J Leukoc Biol.

[21] Chigaev A, Blenc AM, Braaten JV, Kumaraswamy N, Kepley CL, Andrews RP, Oliver JM, Edwards BS, Prossnitz ER, Larson RS, Sklar LA (2001). Real time analysis of the affinity regulation of alpha 4-integrin. The physiologically activated receptor is intermediate in affinity between resting and Mn(2+) or antibody activation. J Biol Chem.

[22] Gattei V, Bulian P, Del Principe MI, Zucchetto A, Maurillo L, Buccisano F, Bomben R, Dal-Bo M, Luciano F, Rossi FM, Degan M, Amadori S, Del Poeta G (2008). Relevance of CD49d protein expression as overall survival and progressive disease prognosticator in chronic lymphocytic leukemia. Blood.

[23] Gupta SK, Pillarisetti K, Thomas RA, Aiyar N (2001). Pharmacological evidence for complex and multiple site interaction of CXCR4 with SDF-1alpha: implications for development of selective CXCR4 antagonists. Immunol Lett.

[24] Hoellenriegel J, Zboralski D, Maasch C, Rosin NY, Wierda WG, Keating MJ, Kruschinski A, Burger JA (2014). The Spiegelmer NOX-A12, a novel CXCL12 inhibitor, interferes with chronic lymphocytic leukemia cell motility and causes chemosensitization. Blood.

[25] Chigaev A, Waller A, Amit O, Halip L, Bologa CG, Sklar LA (2009). Real-time analysis of conformation-sensitive antibody binding provides new insights into integrin conformational regulation. J Biol Chem.

[26] Balatti V, Bottoni A, Palamarchuk A, Alder H, Rassenti LZ, Kipps TJ, Pekarsky Y, Croce CM (2012). NOTCH1 mutations in CLL associated with trisomy 12. Blood.

[27] Lapidot T, Dar A, Kollet O (2005). How do stem cells find their way home?. Blood.

[28] Seitz G, Boehmler AM, Kanz L, Mohle R (2005). The role of sphingosine 1-phosphate receptors in the trafficking of hematopoietic progenitor cells. Ann N Y Acad Sci.

[29] Ratajczak MZ, Lee H, Wysoczynski M, Wan W, Marlicz W, Laughlin MJ, Kucia M, Janowska-Wieczorek A, Ratajczak J (2010). Novel insight into stem cell mobilization-plasma sphingosine-1-phosphate is a major chemoattractant that directs the egress of hematopoietic stem progenitor cells from the bone marrow and its level in peripheral blood increases during mobilization due to activation of complement cascade/membrane attack complex. Leukemia.

[30] Granado MH, Gangoiti P, Ouro A, Arana L, Gonzalez M, Trueba M, Gomez-Munoz A (2009). Ceramide 1-phosphate (C1P) promotes cell migration Involvement of a specific C1P receptor. Cellular signalling.

[31] Arana L, Gangoiti P, Ouro A, Trueba M, Gomez-Munoz A (2010). Ceramide and ceramide 1-phosphate in health and disease. Lipids Health Dis.

[32] Lemoli RM, Ferrari D, Fogli M, Rossi L, Pizzirani C, Forchap S, Chiozzi P, Vaselli D, Bertolini F, Foutz T, Aluigi M, Baccarani M, Di Virgilio F (2004). Extracellular nucleotides are potent stimulators of human hematopoietic stem cells *in vitro* and *in vivo*. Blood.

[33] Rossi L, Manfredini R, Bertolini F, Ferrari D, Fogli M, Zini R, Salati S, Salvestrini V, Gulinelli S, Adinolfi E, Ferrari S, Di Virgilio F, Baccarani M, Lemoli RM (2007). The extracellular nucleotide UTP is a potent inducer of hematopoietic stem cell migration. Blood.

[34] Riches JC, O'Donovan CJ, Kingdon SJ, McClanahan F, Clear AJ, Neuberg DS, Werner L, Croce CM, Ramsay AG, Rassenti LZ, Kipps TJ, Gribben JG (2014). Trisomy 12 chronic lymphocytic leukemia cells exhibit upregulation of integrin signaling that is modulated by NOTCH1 mutations. Blood.

[35] Honczarenko M, Douglas RS, Mathias C, Lee B, Ratajczak MZ, Silberstein LE (1999). SDF-1 responsiveness does not correlate with CXCR4 expression levels of developing human bone marrow B cells. Blood.

[36] Quiroga MP, Burger JA (2010). BCR-mediated decrease of CXCR4 and CD62L in CLL. Cancer research.

[37] Quiroga MP, Balakrishnan K, Kurtova AV, Sivina M, Keating MJ, Wierda WG, Gandhi V, Burger JA (2009). B-cell antigen receptor signaling enhances chronic lymphocytic leukemia cell migration and survival: specific targeting with a novel spleen tyrosine kinase inhibitor, R406. Blood.

[38] Bannard O, Horton RM, Allen CD, An J, Nagasawa T, Cyster JG (2013). Germinal center centroblasts transition to a centrocyte phenotype according to a timed program and depend on the dark zone for effective selection. Immunity.

[39] Yoshida N, Kitayama D, Arima M, Sakamoto A, Inamine A, Watanabe-Takano H, Hatano M, Koike T, Tokuhisa T (2011). CXCR4 expression on activated B cells is downregulated by CD63 and IL-21. J Immunol.

[40] Calissano C, Damle RN, Marsilio S, Yan XJ, Yancopoulos S, Hayes G, Emson C, Murphy EJ, Hellerstein MK, Sison C, Kaufman MS, Kolitz JE, Allen SL, Rai KR, Ivanovic I, Dozmorov IM (2011). Intraclonal complexity in chronic lymphocytic leukemia: fractions enriched in recently born/divided and older/quiescent cells. Mol Med.

[41] Shulman Z, Pasvolsky R, Woolf E, Grabovsky V, Feigelson SW, Erez N, Fukui Y, Alon R (2006). DOCK2 regulates chemokine-triggered lateral lymphocyte motility but not transendothelial migration. Blood.

[42] Woolf E, Grigorova I, Sagiv A, Grabovsky V, Feigelson SW, Shulman Z, Hartmann T, Sixt M, Cyster JG, Alon R (2007). Lymph node chemokines promote sustained T lymphocyte motility without triggering stable integrin adhesiveness in the absence of shear forces. Nat Immunol.

[43] Del Giudice I, Rossi D, Chiaretti S, Marinelli M, Tavolaro S, Gabrielli S, Laurenti L, Marasca R, Rasi S, Fangazio M, Guarini A, Gaidano G, Foa R (2012). NOTCH1 mutations in +12 chronic lymphocytic leukemia (CLL) confer an unfavorable prognosis, induce a distinctive transcriptional profiling and refine the intermediate prognosis of +12 CLL. Haematologica.

[44] Rossi D, Rasi S, Fabbri G, Spina V, Fangazio M, Forconi F, Marasca R, Laurenti L, Bruscaggin A, Cerri M, Monti S, Cresta S, Fama R, De Paoli L, Bulian P, Gattei V (2012). Mutations of NOTCH1 are an independent predictor of survival in chronic lymphocytic leukemia. Blood.

[45] Fabbri G, Rasi S, Rossi D, Trifonov V, Khiabanian H, Ma J, Grunn A, Fangazio M, Capello D, Monti S, Cresta S, Gargiulo E, Forconi F, Guarini A, Arcaini L, Paulli M (2011). Analysis of the chronic lymphocytic leukemia coding genome: role of NOTCH1 mutational activation. J Exp Med.

[46] Puente XS, Pinyol M, Quesada V, Conde L, Ordonez GR, Villamor N, Escaramis G, Jares P, Bea S, Gonzalez-Diaz M, Bassaganyas L, Baumann T, Juan M, Lopez-Guerra M, Colomer D, Tubio JM (2011). Whole-genome sequencing identifies recurrent mutations in chronic lymphocytic leukaemia. Nature.

[47] Fabbri G, Khiabanian H, Holmes AB, Wang J, Messina M, Mullighan CG, Pasqualucci L, Rabadan R, Dalla-Favera R (2013). Genetic lesions associated with chronic lymphocytic leukemia transformation to Richter syndrome. J Exp Med.

[48] Santos FP, O'Brien S (2012). Small lymphocytic lymphoma and chronic lymphocytic leukemia: are they the same disease?. Cancer J.

[49] Thompson PA, Ferrajoli A, O'Brien S, Wierda WG, Keating MJ, Burger JA (2014). Trisomy 12 is associated with an abbreviated redistribution lymphocytosis during treatment with the BTK inhibitor ibrutinib in patients with chronic lymphocytic leukaemia. British journal of haematology.

