# Supplementary material for: CXCL12-induced VLA-4 activation is impaired in trisomy 12 chronic lymphocytic leukemia cells: a role for CCL21
Source: Oncotarget. 2015 Mar 26;6(14):12048–60. doi: 10.18632/oncotarget.3660 (PMC4494922; doi:10.18632/oncotarget.3660)
Supplement: Supplementary file 1 [file oncotarget-06-12048-s001.pdf]

CXCL12-induced VLA-4 activation is impaired in trisomy 12 chronic lymphocytic leukemia cells: a role for CCL21

Supplementary Material

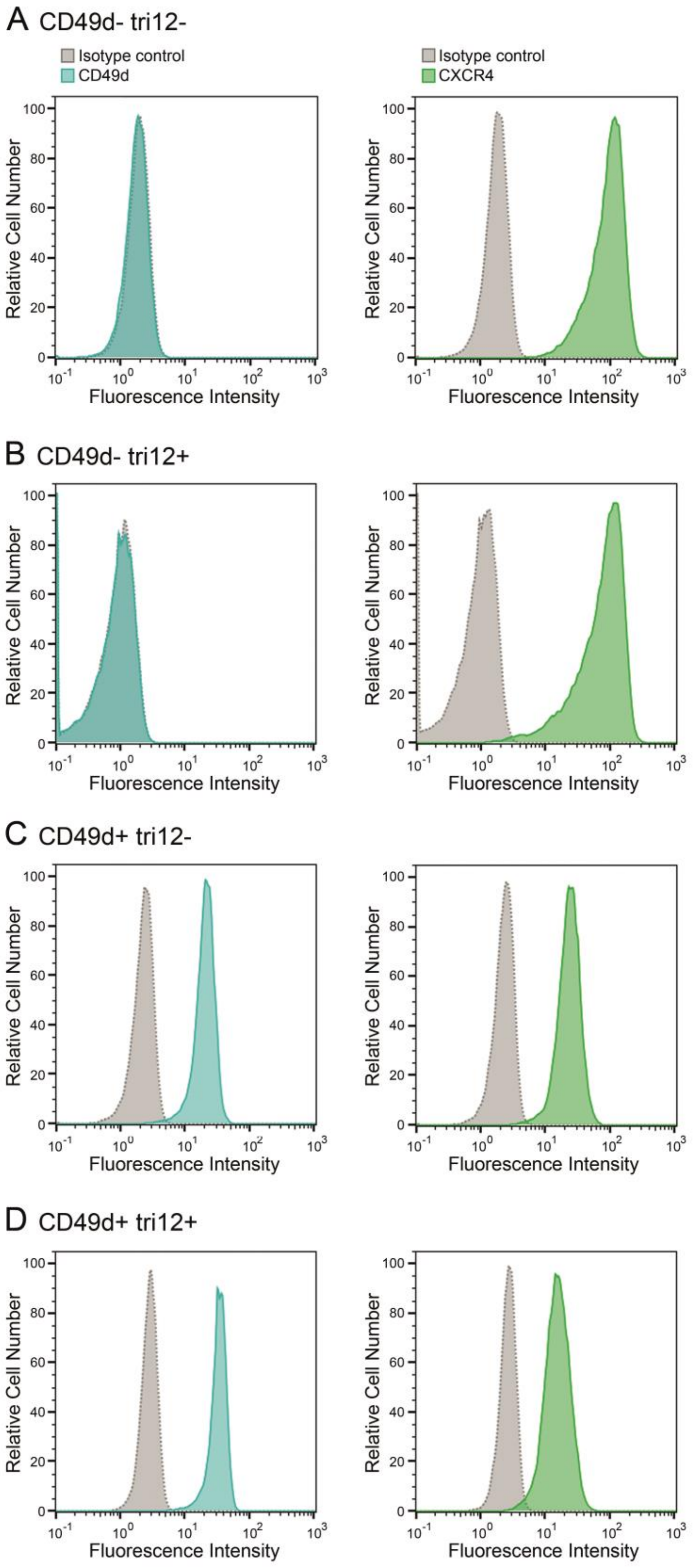

Supplemental Figure 1: CD49d and CXCR4 expression are inverse in CLL. (A-D) Cytometrical histograms of CD49d (*left*, blue) and CXCR4 (*right*, green) expression (MFI values) and respective isotype controls (grey) of individual CLL samples representative for the four subgroups defined by the CD49d status and the presence of tri12.

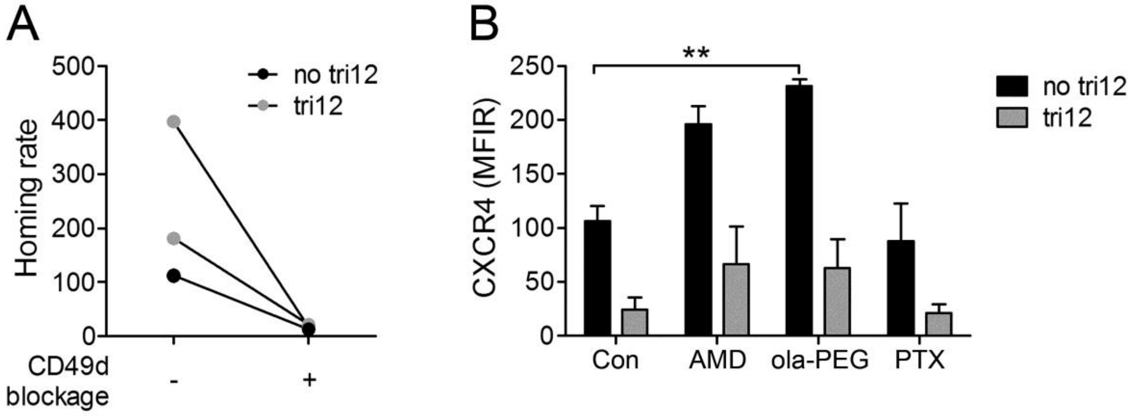

Supplemental figure 2: No tri12 as well as tri12 CLL cells home VLA-4-dependent to BM and homed no tri12 CLL cells display an upregulated CXCR4 expression. (A) Bone marrow homing rates of no tri12 and tri12 CLL cells upon treatment with the anti-CD49d mAb HP2.1. Homing rates were normalized as described [8, 9]: number of human cells analyzed per 10^6 mouse cells (total cells) per 10^6 injected viable human target cells. Each of the three depicted experiments has been performed in duplicates. (B) Cytometrical determination of CXCR4 expression (MFIR) of human CD5+CD19+ no tri12 and tri12 CLL cells that had homed under the indicated treatment regimes to murine BM. The columns show the mean and SD of three different no tri12 and four different tri12 patient samples. Each experiment has been performed in duplicates. Statistical significance refers to the paired Student *t* test.

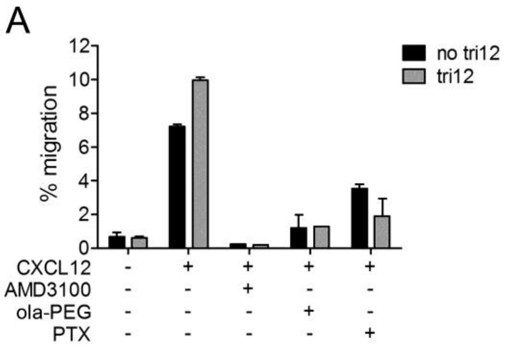

Supplemental figure 3: Chemotactic activity of no tri12 and tri12 CLL cells towards CXCL12 is retained and CXCR4/CXCL12 specific. Chemotaxis assays were performed for 2 hours using 5 µm pore Boyden chamber assays. Cells, pretreated with PTX (100 ng/ml) or AMD3100 (5 µM), where indicated, were added in the upper chamber, CXCL12 (100 ng/µl) and NOX-A12 (100 nM), where indicated, were present in the lower chamber. Migrated CLL cells were defined and counted as described in Material and Methods. The data represent one out of three independent experiments each; the columns show the mean and SD of duplicates.

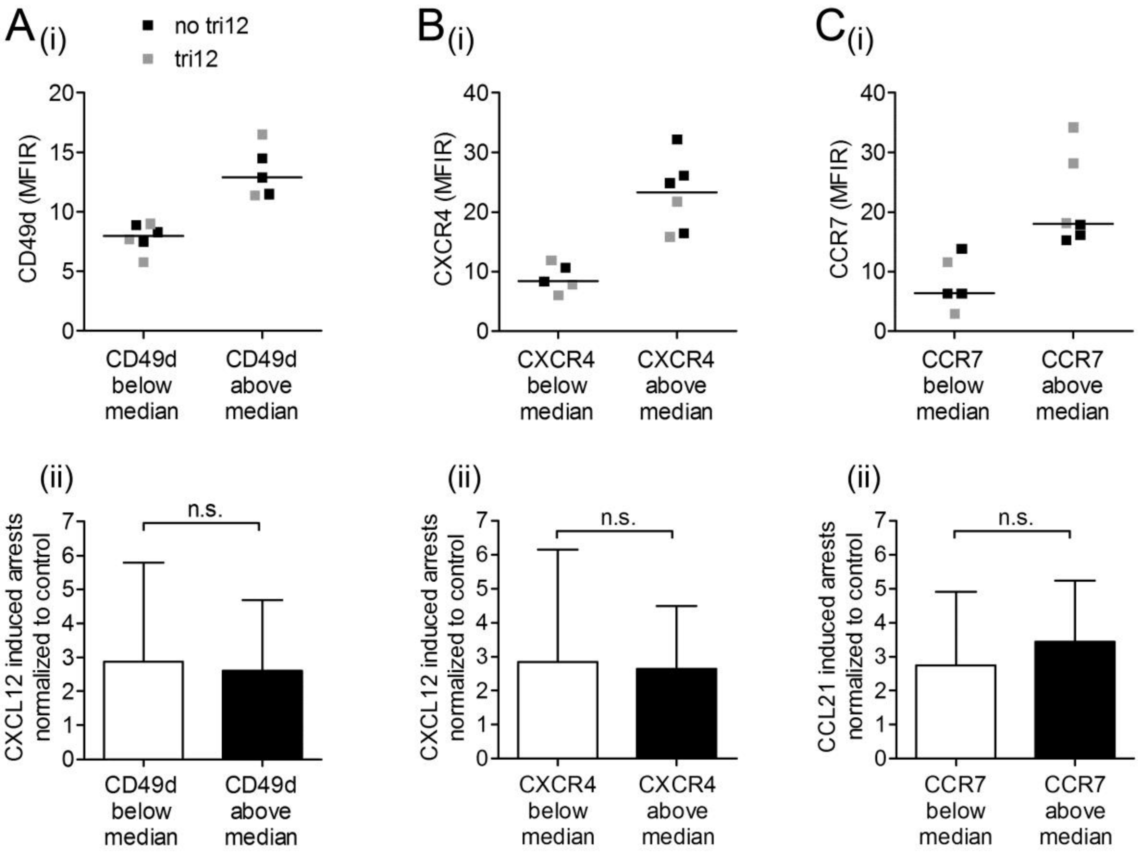

Supplemental figure 4: Variations in CD49d, CXCR4 and CCR7 surface expression do not alter CXCL12- or CCL21-induced arrests of CD49d+ CLL cells on VCAM-1. (A) Control analysis to Figure 3B: (i) CD49d surface expression levels of no tri12 and tri12 CLL samples used in Figure 3B were split into groups with low (n = 6) or high (n = 5) CD49d levels according to the median CD49d expression. Tri12 CLL samples are marked in grey, no tri12 CLL cells in black. (ii) CLL samples were grouped using the median CD49d expression as the cutoff, and total arrests to CXCL12/VCAM-1 were normalized to VCAM-1 controls. (B) Control analysis to Figure 3B: (i) CXCR4 surface expression levels of no tri12 and tri12 CLL samples used in Figure 3B were split into groups with low (n = 5) or high (n = 6) CXCR4 levels according to the median CXCR4 expression. Tri12 CLL samples are marked in grey, as described above. (ii) CLL samples were grouped using the median CXCR4 expression as the cutoff, and total arrests to CXCL12/VCAM-1 were normalized to VCAM-1 controls. (C) Control analysis to Figure 6B: (i) CCR7 surface expression levels of no tri12 and

tri12 CLL samples used in Figure 6B were split into groups with low (n = 5) or high (n = 6) CD49d levels according to the median CCR7 expression. Tri12 CLL samples are marked in grey, as described above. (ii) CLL samples were grouped using the median CCR7 expression as the cutoff, and total arrests to CCL21/VCAM-1 were normalized to VCAM-1 controls. Scatter plots, the middle line indicates the median. Columns represent the mean ± SD.

Supplemental Table 1: Detailed patient characteristics. Rai stage was defined according to Rai *et al*. [1] Treatment status: CD49d expression (cutoff 30%) was analyzed as described [2, 3] in patients that were either chemonaive or not treated within the last 6 months. The mutational status of the IgVH genes was defined by the percentage of sequence homology with the germline equivalent (M, mutated, <98% homology; UM, unmutated, ≥98% homology). ZAP-70 expression was evaluated as recommended [4] (low, NKT/B ratio >3.5; high, NKT/B ratio <3.1). CD38 status was defined as described [5] (low, <30%; high, ≥30%). Chromosomal aberrations were determined according to Döhner *et al*. [6] Notch-1 mutational status was defined by amplification refractory mutation system (ARMS) PCR according to Rossi *et al*. [7]. ND, not determined.

Supplemental Table 2: Subgroup analysis of the Notch-1 mutational status of no tri12 and tri12 CLL cells. Clinical and prognostic markers were analyzed as described in the legend to Supplemental Table 1. Notch-1 mutational status was defined by amplification refractory mutation system (ARMS) PCR according to Rossi *et al*. [7]. ND, not determined.

References

1. Rai KR, Sawitsky A, Cronkite EP, Chanana AD, Levy RN and Pasternack BS. Clinical staging of chronic lymphocytic leukemia. Blood. 1975; 46(2):219-234.

2. Bulian P, Shanafelt TD, Fegan C, Zucchetto A, Cro L, Nuckel H, Baldini L, Kurtova AV, Ferrajoli A, Burger JA, Gaidano G, Del Poeta G, Pepper C, Rossi D and Gattei V. CD49d is the strongest flow cytometry-based predictor of overall survival in chronic lymphocytic leukemia. J Clin Oncol. 2014; 32(9):897-904.

3. Gattei V, Bulian P, Del Principe MI, Zucchetto A, Maurillo L, Buccisano F, Bomben R, Dal-Bo M, Luciano F, Rossi FM, Degan M, Amadori S and Del Poeta G. Relevance of CD49d protein expression as overall survival and progressive disease prognosticator in chronic lymphocytic leukemia. Blood. 2008; 111(2):865-873.

4. Letestu R, Rawstron A, Ghia P, Villamor N, Boeckx N, Boettcher S, Buhl AM, Duerig J, Ibbotson R, Kroeber A, Langerak A, Le Garff-Tavernier M, Mockridge I, Morilla A, Padmore R, Rassenti L, et al. Evaluation of ZAP-70 expression by flow cytometry in chronic lymphocytic leukemia: A multicentric international harmonization process. Cytometry B Clin Cytom. 2006; 70(4):309-314.

5. Damle RN, Wasil T, Fais F, Ghiotto F, Valetto A, Allen SL, Buchbinder A, Budman D, Dittmar K, Kolitz J, Lichtman SM, Schulman P, Vinciguerra VP, Rai KR, Ferrarini M and

Chiorazzi N. Ig V gene mutation status and CD38 expression as novel prognostic indicators in chronic lymphocytic leukemia. Blood. 1999; 94(6):1840-1847.

6. Dohner H, Stilgenbauer S, Benner A, Leupolt E, Krober A, Bullinger L, Dohner K, Bentz M and Lichter P. Genomic aberrations and survival in chronic lymphocytic leukemia. The New England journal of medicine. 2000; 343(26):1910-1916.

7. Rossi D, Rasi S, Fabbri G, Spina V, Fangazio M, Forconi F, Marasca R, Laurenti L, Bruscaggin A, Cerri M, Monti S, Cresta S, Fama R, De Paoli L, Bulian P, Gattei V, et al. Mutations of NOTCH1 are an independent predictor of survival in chronic lymphocytic leukemia. Blood. 2012; 119(2):521-529.

8. Brachtl G, Sahakyan K, Denk U, Girbl T, Alinger B, Hofbauer SW, Neureiter D, Hofbauer JP, Egle A, Greil R and Hartmann TN. Differential bone marrow homing capacity of VLA-4 and CD38 high expressing chronic lymphocytic leukemia cells. PLoS One. 2011; 6(8):e23758.

9. Hartmann TN, Grabovsky V, Wang W, Desch P, Rubenzer G, Wollner S, Binsky I, Vallon-Eberhard A, Sapoznikov A, Burger M, Shachar I, Haran M, Honczarenko M, Greil R and Alon R. Circulating B-cell chronic lymphocytic leukemia cells display impaired migration to lymph nodes and bone marrow. Cancer Res. 2009; 69(7):3121-3130.
